# Antibiotic consumption and time to recovery from uncomplicated urinary tract infection: secondary analysis of observational data from a point-of-care test trial

**DOI:** 10.3399/BJGP.2022.0011

**Published:** 2022-11-15

**Authors:** Amal Gadalla, Hannah Wise, Daniel Farewell, Kathryn Hughes, Carl Llor, Michael Moore, Theo JM Verheij, Paul Little, Christopher C Butler, Nick A Francis

**Affiliations:** Division of Population Medicine;; Division of Population Medicine;; Division of Population Medicine;; PRIME Centre Wales, Division of Population Medicine, Cardiff University, Cardiff, UK.; Department of Public Health, University of Southern Denmark, Denmark; University Institute in Primary Care Research Jordi Gol, Via Roma Health Centre, Barcelona, Spain.; School of Primary Care, Population Sciences and Medical Education, University of Southampton, Southampton, UK.; Julius Center for Health Sciences and Primary Care, University Medical Center Utrecht, Utrecht, the Netherlands.; School of Primary Care, Population Sciences and Medical Education, University of Southampton, Southampton, UK.; Nuffield Department of Primary Care Health Sciences, University of Oxford, Oxford, UK.; Division of Population Medicine, School of Medicine, Cardiff University, Cardiff, UK; School of Primary Care, Population Sciences and Medical Education, University of Southampton, Southampton, UK.

**Keywords:** anti-bacterial agents, antibiotic consumption, general practice, recovery without antibiotics, uncomplicated, urinary tract infections

## Abstract

**Background:**

Randomised trials provide high-quality evidence on the effects of prescribing antibiotics for urinary tract infection (UTI) but may not reflect the effects in those who consume antibiotics. Moreover, they mostly compare different antibiotic types or regimens but rarely include a ‘no antibiotic’ group.

**Aim:**

To estimate the effect of antibiotic consumption, rather than prescription, on time to recovery in females with uncomplicated UTI.

**Design and setting:**

Secondary analysis of 14-day observational data from a point-of-care test trial for UTI in primary care in England, the Netherlands, Spain, and Wales, which ran from 2012 to 2014. Clinicians treated patients using their own judgement, providing immediate, delayed, or no antibiotic.

**Method:**

UTI-symptomatic females who either consumed or did not consume antibiotics during a 14-day follow-up were included. Antibiotic consumption was standardised across participants and grouped into either ≤3 or >3 standardised antibiotic days. To account for confounders, a robust propensity score matching analysis was conducted. Adjusted Kaplan–Meier and Cox proportional hazard models were employed to estimate time to recovery and hazard ratios, respectively.

**Results:**

A total of *n* = 333 females who consumed antibiotics and *n* = 80 females who did not consume antibiotics were identified and included in the study. The adjusted median time to recovery was 2 days longer among patients who did not consume antibiotics (9 days, 95% confidence interval [CI] = 7 to 12) compared with those who did (7 days, 95% CI = 7 to 8). No difference was found between those who consumed ≤3 (7 days, 95% CI = 7 to 8) compared with >3 standardised antibiotic days (7 days, 95% CI = 6 to 9).

**Conclusion:**

Consuming antibiotics was associated with a reduction in self-reported time to recovery, but more antibiotics exposure was not associated with faster recovery in this study.

## INTRODUCTION

Uncomplicated urinary tract infection (UTI) is common among females, with symptoms lasting 10 days on average.^1^^,^^2^ The infection places a huge burden on individuals and healthcare systems,^3^^–^^6^ and contributes significantly to antibiotic prescribing in the community.

Uncomplicated UTI is treated with empirical antibiotics that vary in type and duration within and across countries.^7^^,^^8^ The majority of patients respond adequately to these treatments and some patients choose not to use antibiotics.^9^^–^^12^ However, the incidence of antibiotic-resistant UTI is increasing.^13^ Guidelines primarily use evidence from trials that seldom compare antibiotic use with no antibiotic use and rarely take account of antibiotic consumption as opposed to antibiotic prescription.^14^

This study aims to report the effect of antibiotic consumption compared with no antibiotic consumption (rather than prescription) and the consumed antibiotic amount, on time to patient-reported recovery in females with uncomplicated UTI.

## METHOD

Data from the Point of care testing for urinary tract infection in primary care (POETIC) trial were analysed.^15^^,^^16^ The trial ran from 2012 to 2014 and involved 43 general practices in England, the Netherlands, Spain, and Wales. Non-pregnant, adult female participants who had at least one of the main uncomplicated UTI symptoms — dysuria, frequency, or urgency — were recruited during routine consultation. Patients with pyelonephritis, other severe systemic symptoms, or who received antibiotics 4 weeks before recruitment, were on long-term antibiotics, or had genitourinary tract abnormalities were not recruited.^15^ Clinicians treated patients (providing immediate, delayed, or no antibiotic at all) using their own judgement or with the aid of the Flexicult™, which provided results on pathogen and antibiotic strength, mg × total number of consumed doses published defined daily dose (DDD)^19^ antibiotics sensitivity 24 hours after recruitment, and, if necessary, treatment was changed accordingly.^15^

**Table:** How this fits in

Limited evidence from randomised trials suggests that, on average, prescribing antibiotics improves recovery in females with uncomplicated urinary tract infection (UTI), and that short (3-day) courses are as effective as longer courses. However, not all antibiotic prescriptions are consumed, and some females recover without antibiotics. Therefore, it is important to explore the relationship between antibiotic consumption and time to recovery. Adjusting for various confounders, this study found that females with UTI who consumed antibiotics recovered faster than those who were not prescribed or did not consume antibiotics. This study also found no difference in recovery time among those who consumed more antibiotics. However, those who consumed no antibiotics and did not completely recover reported only mild symptoms by the estimated recovery time for their group.

Clinicians completed a baseline questionnaire detailing the severity of symptoms, history of previous UTI treatments, and management chosen. Patients then completed a 14-day diary in which they rated the severity of 11 symptoms:

- urgency;
- burning or pain when passing urine (dysuria);
- daytime frequency;
- night-time frequency;
- smelly urine;
- pain in the side;
- abdominal pain;
- fever;
- blood in urine;
- restricted activity; and
- general unwell feeling on a scale of 0 to 6 (0 = ‘not affected’ and 6 = ‘as bad as can be’).

Daily consumption of medication and day of recovery (patient answered a direct question on when they have felt completely recovered) were also documented.

### Data inclusion

Data from patients who were either prescribed an antibiotic immediately, or not at all, were included. Patients with delayed or changed antibiotic because of resistant infection were excluded to create a clear definition of both exposure and outcome. Patients with missing data for the type, strength, and dose of antibiotic used were excluded.

Patients were categorised into two groups: whether they consumed any antibiotic during follow-up or not. A standardised antibiotic consumption unit (standardised antibiotic days) was implemented to allow comparison between different antibiotic strengths and dosing regimens.^17^^–^^19^ Consumption ranged from 0.17 to 14 standardised antibiotic days and patients who consumed antibiotics were categorised into two categories: ≤3 and >3 standardised antibiotic days:

$$\frac{\text{antibiotic}\;\text{strength},\;\text{mg}\times \text{total}\;\text{number}\;\text{of}\;\text{consumed}\;\text{doses}}{\text{published}\;\text{defined}\;\text{daily}\;\text{dose}\;{\left(\text{DDD}\right)}^{19}}$$

### Statistical analysis

Kaplan–Meier survival analysis^20^ and Cox proportional hazard models double robust method^21^^,^^22^ were used to compare median time with recovery and hazard ratio (HR), respectively, between patients who consumed antibiotics and those who did not, and between those who consumed >3 and ≤3 standardised antibiotic days.

Propensity score matching models were used to adjust for confounding factors including the severity scores for baseline symptoms (listed above), analgesic use (ibuprofen, paracetamol, co-codamol, metamizole, and tramadol), antifungal use (clotrimazole oral, topical and pessaries, and fluconazole), antimuscarinic use (solifenacin, trospium chloride, and mebeverine hydrochloride), age, and country. For the analyses where standardised antibiotic days were compared, day-3 symptom scores were added as confounding factors, as this might have also affected patients’ decisions to continue taking their antibiotics and it did improve the propensity score balance between the groups (data not shown).

Among many attempted propensity score matching methods, marginal mean weighting through stratification^23^ yielded a good balance using the R package ‘MatchIT’.^24^ In this method, propensity score was estimated using a logistic regression of the antibiotic consumption on the confounding factors listed above. The researchers used all observations from groups and no units were discarded during matching.

Sensitivity analysis was conducted to include UTI treatment history in the calculation of the propensity score. Data analysis was conducted in R (version 4.0.5).

## RESULTS

### Antibiotic consumption

**Figure 1.**
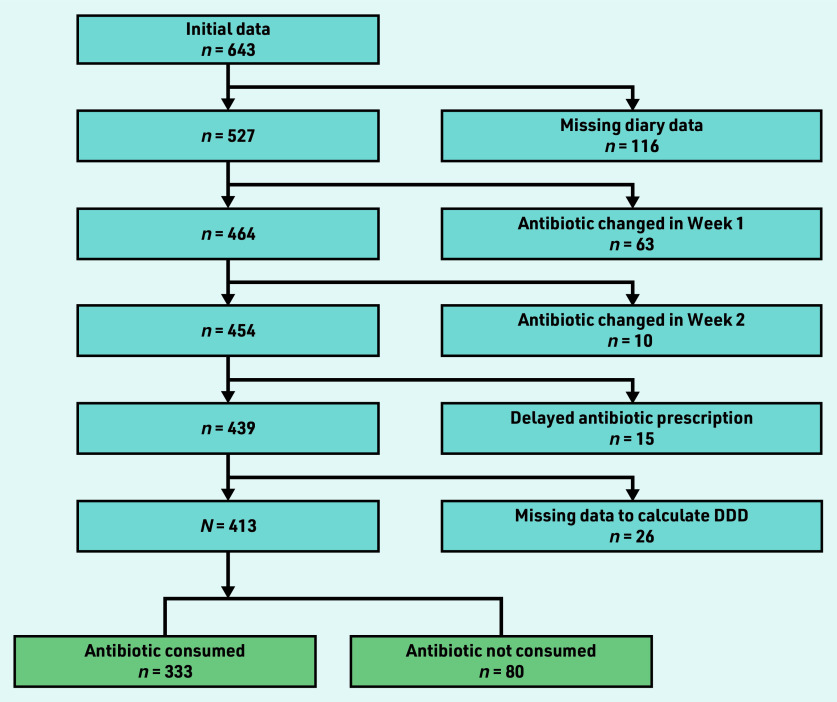
*Flow chart showing application of the exclusion criteria. DDD = defined daily dose.*

The POETIC cohort consisted of 643 patients with full baseline data. The final cohort for this study included *N* = 413 (64.2%) patients after exclusion of patients with missing follow-up data (*n* = 116, 18.0%), changed antibiotics (*n* = 73, 11.4%), delayed antibiotics (*n* = 15, 2.3%), and missing data required to calculate the standardised antibiotic consumption (*n* = 26, 4.0%) (Figure 1).

Of the final cohort, *n* = 80 (19.4%) patients consumed no antibiotics, of whom *n* = 75 were not prescribed antibiotics and *n* = 5 were prescribed antibiotics but did not consume any. Among those who consumed antibiotics (*n* = 333, 80.6%), nine antibiotics were used: trimethoprim (*n* = 146, 43.8%), nitrofurantoin (*n* = 72, 21.6%), fosfomycin (*n* = 63, 18.9%), ciprofloxacin (*n* = 13, 3.9%), norfloxacin (*n* = 13, 3.9%), amoxicillin clavulanic (*n* = 9, 2.7%), amoxicillin (*n* = 8, 2.4%), cephalexin (*n* = 8, 2.4%), and cefuroxime (*n* = 1, 0.3%). Among those who consumed ≤3 standardised antibiotic days (*n* = 201), *n* = 181 (90.0%) patients were prescribed a course of 1 to 3 days, *n* = 17 (8.5%) patients were prescribed a 5- or 7-day course, and *n* = 3 (1.5%) patients had missing data for prescription duration. Among those who consumed >3 standardised antibiotic days (*n* = 132), there were *n* = 11 (8.3%) patients with 1 to 3-day prescription and *n* = 121 (91.7%) with 4 to 8 or a 10-day prescription.

### Potential confounders

The mean age of patients was 48.4 (95% confidence interval [CI] = 47.4 to 50.9) years for the entire cohort, and similarly distributed across the antibiotic consumption groups (Table 1). Patients were from four nations, with 31.2% (129/413) from England, 33.4% (138/413) from Wales, 28.3% (117/413) from Spain, and 7.0% (29/413) from Netherlands.

**Table 1.** Baseline characteristics of study group, *N*= 413

**Characteristic**	**Antibiotic consumption groups**			

	**Not consumed (*n*= 80)^a^**	**Consumed (*n*= 333)^a^**	**Within antibiotic consumption**	

			**≤3 Standardised antibiotic days (*n*= 201)^a^**	**>3 Standardised antibiotic days (*n*= 132)^a^**
**Age**				
Mean, years (95% CI)	47.1 (43.1 to 51.1)	48.7 (46.7 to 50.6)	47.4 (44.8 to 50.0)	50.7 (47.7 to 53.6)

**Country**				
*n* (%) within antibiotic consumption				
Netherlands	7 (8.7)	22 (6.6)	2 (1.0)	20 (15.2)
England	26 (32.5)	103 (30.9)	78 (38.8)	25 (18.9)
Wales	20 (25.0)	118 (35.5)	60 (29.9)	58 (43.9)
Spain	27 (33.8)	90 (27.0)	61 (30.3)	29 (22.0)

**Severity of the initial symptoms**				
Mean (95% CI of score [0–6])				
Urgency	3.01 (2.57 to 3.45)	3.54 (3.35 to 3.73)	3.56 (3.31 to 3.81)	3.52 (3.22 to 3.82)
Dysuria	2.79 (2.34 to 3.25)	3.10 (2.89 to 3.32)	3.16 (2.89 to 3.44)	3.01 (2.67 to 3.35)
Daytime frequency	3.46 (3.07 to 3.85)	3.66 (3.48 to 3.85)	3.63 (3.38 to 3.88)	3.72 (3.43 to 4.00)
Night-time frequency	2.64 (2.18 to 3.10)	3.00 (2.79 to 3.21)	3.01 (2.73 to 3.29)	2.98 (2.68 to 3.29)
Smelly urine	1.58 (1.16 to 2.00)	1.90 (1.68 to 2.11)	1.82 (1.55 to 2.09)	2.02 (1.66 to 2.38)
Pain in the side	1.37 (0.95 to 1.79)	1.29 (1.10 to 1.48)	1.22 (0.98 to 1.47)	1.39 (1.07 to 1.70)
Abdominal pain	1.74 (1.33 to 2.15)	2.00 (1.80 to 2.20)	2.01 (1.74 to 2.28)	1.98 (1.67 to 2.30)
Fever	0.46 (0.20 to 0.72)	0.80 (0.65 to 0.96)	0.84 (0.64 to 1.04)	0.75 (0.50 to 0.99)
Blood in urine	0.41 (0.16 to 0.65)	0.70 (0.55 to 0.86)	0.73 (0.53 to 0.94)	0.66 (0.43 to 0.90)
Restricted activities	1.41 (1.02 to 1.80)	1.55 (1.34 to 1.75)	1.52 (1.25 to 1.78)	1.59 (1.26 to 1.92)
Generally unwell	1.74 (1.32 to 2.16)	2.14 (1.95 to 2.34)	2.13 (1.88 to 2.38)	2.17 (1.85 to 2.49)

**UTI treatment within the past year^b^**				
*n* (%) within antibiotic consumption groups				
No	16 (29.1)	93 (33.9)	57 (34.5)	36 (33.0)
Yes	39 (70.9)	181 (66.1)	108 (65.5)	73 (67.0)

**Antifungal use^c^**				
*n* (%) within antibiotic consumption groups				
No	78 (97.5)	327 (98.2)	197 (98.0)	130 (98.5)
Yes	2 (2.5)	6 (1.8)	4 (2.0)	2 (1.5))

**Antimuscarinic use^d^**				
*n* (%)				
No	79 (98.7)	330 (99.1)	199 (99.0)	131 (99.2)
Yes	1 (1.3)	3 (0.9)	2 (1.0)	1 (0.8)

**Analgesic use^e^**				
*n* (%) within antibiotic consumption				
No	72 (90.0)	318 (95.5)	192 (95.5)	126 (95.5)
Yes	8 (10.0)	15 (4.5)	9 (4.5)	6 (4.5)

**UTI confirmed by culture^f^**				
*n* (%) within antibiotic consumption				
No	55 (74.3)	206 (63.6)	131 (66.8)	75 (58.6)
Yes	19 (25.7)	118 (36.4)	65 (33.2)	53 (41.4)

a *If any of patient’s characteristics contained missing observation they were not included in percentage calculations.*

b *For UTI treatment within the past year and UTI microbiology results there were 84 and 15 missing data points, respectively.*

c *Antifungal: clotrimazole pessaries, fluconazole, Canesten combi, and Canesten thrush cream.*

d *Antimuscarinic: Vesicare, Flotros, mebeverinehydrochloride.*

e *Analgesics: ibuprofen, paracetamol, Solpadol, metamizole, tramadol and co-codamol.*

f *Culture for UTI diagnosis was done after antibiotic prescription. UTI = urinary tract infection.*

At baseline, mean symptom severity scores were generally slightly higher among those who consumed antibiotics compared to those who did not (Table 1), but these differences were less clear among antibiotic consumption levels of ≤3 or >3 standardised antibiotic days.

UTI treatment within the past year was received by 181/274 (66.1%) and 39/55 (70.9%) patients among those who did and did not consume antibiotics, respectively (Table 1). There were 108/165 (65.5%) and 73/109 (67.0%) patients with UTI treatment history among those who consumed ≤3 and >3 standardised antibiotic days, respectively.

Only small proportions of the study cohort consumed over-the-counter analgesics, antifungals, and antimuscarinics at some point in the follow-up. However, there was a lower proportion of females taking antifungals (1.8% versus 2.5%), antimuscarinics (0.9% versus 1.3%), or analgesics (4.5% versus 10.0%) among those who consumed antibiotics compared with those who did not. These proportions were similar across the standardised antibiotic days groups (Table 1).

UTI microbiological culture results were known to clinicians after the initial management decision was made, thus it was not included as a confounder. However, the researchers have summarised its proportions among antibiotic consumption groups in Table 1 to provide clinically relevant information about the study cohort.

### Time to recovery

Most participants, *n* = 331 (80.1%), reported complete symptom recovery during the follow-up. Only two (0.5%) reported not having recovered by day 14, but *n* = 80 (19.4%) had missing recovery data, therefore their recovery time was censored. Among those who consumed no antibiotics, *n* = 50 (62.5%) patients reported recovery during the follow-up, while *n* = 281 (84.4%) reported recovery among those who consumed antibiotics.

**Figure 2.**
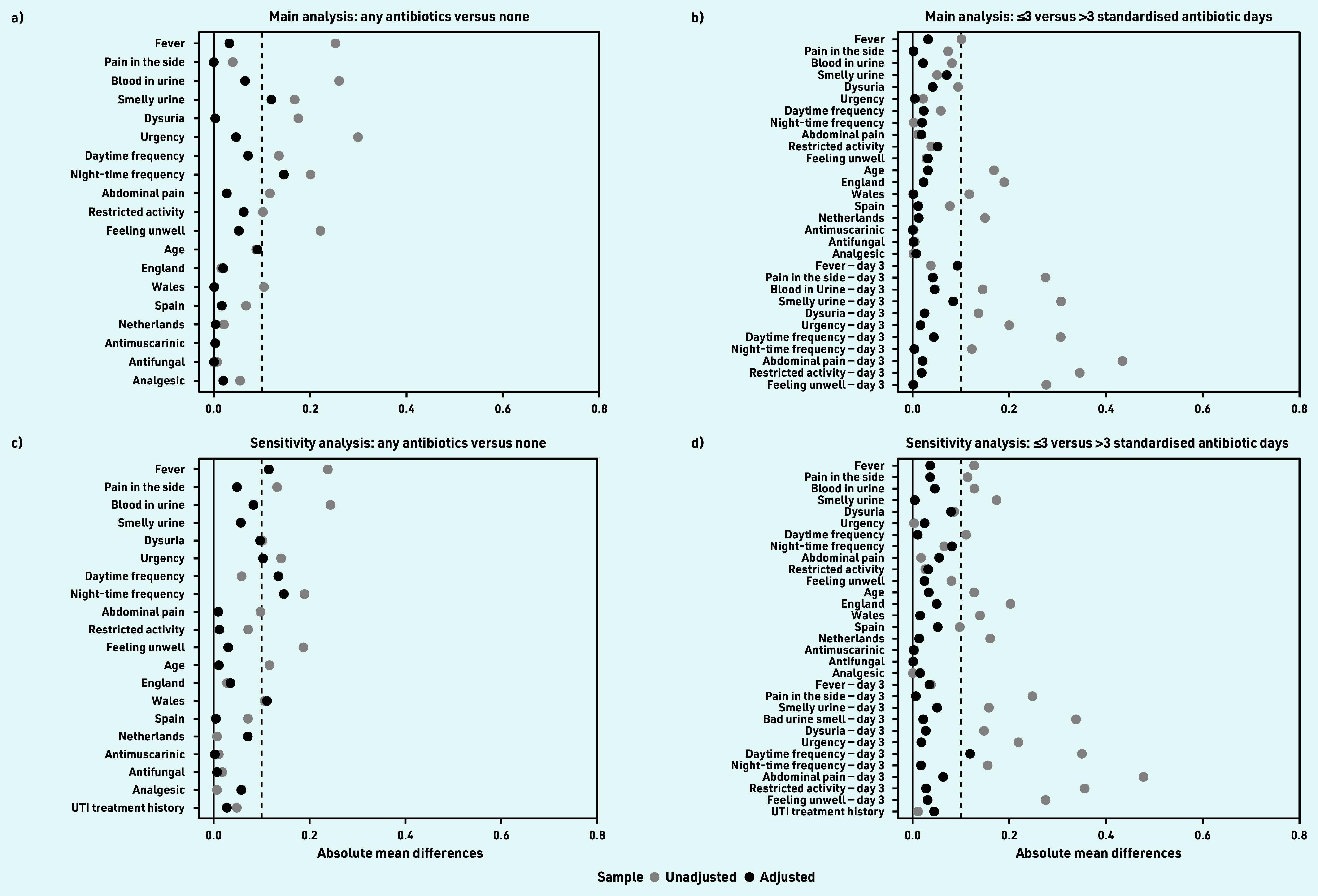
*Covariate balance assessment. The figure shows the absolute standardised mean difference (SMD) in the sample before (grey circle) and after (black circle) propensity score adjustment. SMD is the difference in means of each covariate between antibiotic consumption groups standardised by the pooled standard deviation across both groups. SMDs <0.1 is recommended for prognostically important covariates; however, higher values are acceptable if a double robust method is used as the researchers have implemented in their Cox proportional hazard models.^22^ Panel A shows covariate balance for those who consumed any antibiotics versus those who did not, while panel B is for covariate balance for those consumed ≤3 versus >3 standardised antibiotic days. Panels C and D are for the corresponding sensitivity analyses based on the addition of UTI history. UTI = urinary tract infection.*

**Figure 3.**
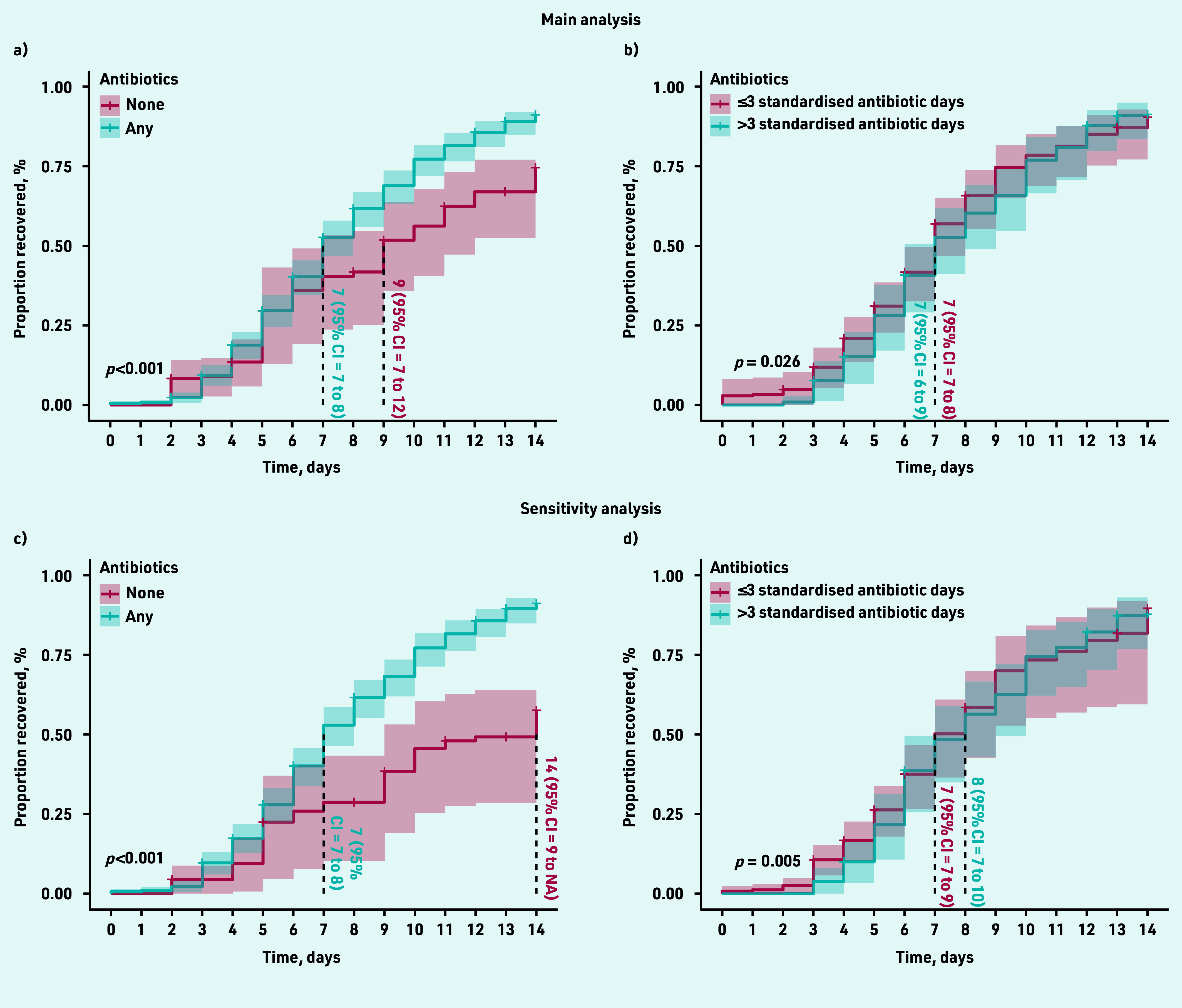
*Propensity score-adjusted survival curves. Figure 3a shows the estimated time to recovery in patients who consumed antibiotics versus those who did not. Figure 3b shows the estimated time to recovery among those who consumed ≤3 standardised antibiotic days compared with those who consumed >3 standardised antibiotic days. Figures 3a and 3b show the main analyses, while Figures 3c and 3d show the sensitivity analyses utilising UTI treatment history. Survival curves were compared using log rank test. Vertical lines from the survival curves demonstrate the median time to recovery. CI = confidence interval. UTI = urinary tract infection.*

The overall estimated median time at which 50% of the cohort reported feeling recovered was 8.0 (95% CI = 7.5 to 8.5) days. Following adequate propensity score balancing (Figure 2), the adjusted estimated median time to recovery was 2 days longer among participants with no antibiotic consumption (9 days, 95% CI = 7 to 12) compared with those who consumed antibiotics (7 days, 95% CI = 7 to 8; log rank test *P*<0.001) (Figure 3A). Those who consumed no antibiotics and had not recovered by day 9 (48/80, 60%) had only mild to moderate average symptom severity scores for the rest of the period (Supplementary Figure S1). The adjusted HR for recovery was 1.72 (95% CI = 1.19 to 2.47) for those who consumed antibiotics compared with those who did not (Table 2).

**Table 2.** Adjusted hazard ratio of recovery among antibiotic consumption levels compared with no antibiotic consumption

**Main analysis**	**Hazard ratio (95% CI)**
**Antibiotic consumption^a^**	
None (*n*= 80)	Referent
Any (*n*= 333)	1.72 (1.19 to 2.47)

≤3 days (*n*= 201)	Referent
>3 days (*n*= 132)	0.90 (0.68 to 1.20)

**Sensitivity analysis^b^**	
None (*n*= 55)	Referent
Any (*n*= 274)	2.65 (1.50 to 4.69)

≤3 days (*n*= 161)	Referent
>3 days (*n*= 102)	0.79 (0.58 to1.09)

a *Antibiotic consumption calculated as number of standardised antibiotic days.*

b *Sensitivity analysis included history of UTI episodes within the past year among the confounders contributed to the propensity score matching.*

The adjusted median time to recovery among those who consumed ≤3 and >3 standardised antibiotic days was similar (7 days for both) with a wider 95% CI among the latter (95% CI = 6 to 9) compared with the former group (95% CI = 7 to 8, log rank test *P* = 0.026) (Figure 3B). This analysis was then repeated excluding those who recovered before or on day 3 to emulate a target trial with similar recovery baseline. Similarly, no difference was seen in the adjusted median time to recovery (both groups were 8 days; 95% CI = 7 to 9, log rank test *P* = 0.086), nor a significant HR (0.90; 95% CI = 0.68 to 1.20) for those who consumed >3 compared to those who consumed ≤3 standardised antibiotic days (Table 2).

### Sensitivity analysis

Patients who reported whether they had received UTI treatment within the last year (*n* = 220) or not (*n* = 109) were included in a sensitivity analysis where this important confounding factor was adjusted for. Both the estimated median time to recovery (Figure 3C) and HR (Table 2) increased among those who did not take antibiotics compared with the main analysis.

Similar to the main analysis, the estimated time to recovery was slightly longer among those who consumed >3 compared with those who consumed ≤3 standardised antibiotic days (Figure 3D). The HR for recovery also did not deviate from the main analysis (Table 2).

## DISCUSSION

### Summary

In this study the researchers found that antibiotic consumption reduced time to recovery from UTI symptoms by 2 days (for a 14-day follow-up) compared with not taking antibiotics, after matching by baseline characteristics and use of other medication. However, when considering history of UTI treatment as a confounder, recovery could be as much as 7 days longer in those who consumed no antibiotics. No substantial difference was found in time to recovery among those who consumed more antibiotics, which was supported by the Cox model HR and target trial analysis.

### Strengths and limitations

Non-adherence is a recognised issue in community infection management,^25^^,^^26^ which weakens conclusions from prescription data. To the authors’ knowledge, only one other study^12^ explicitly explored recovery from UTI using antibiotic consumption rather than prescription data. The present study included most of the antibiotics used to treat uncomplicated UTI, and variable antibiotic exposure (type, dose, and strength) was standardised by calculating a standardised antibiotic consumption. A further strength of this study is the use of propensity score matching based on the initial symptoms to estimate effects on time to recovery. This approach helps correct for indication bias, where those with more severe symptoms are more likely to be prescribed and consume antibiotics. Further, sensitivity analyses using UTI treatment history as a potential confounder were conducted. UTI history is an important factor that affects clinicians’ decisions to prescribe and patients’ decisions to consume antibiotics. However, this information was missing from 84 patients, hence the use of sensitivity analysis. The results highlighted the importance of this confounder and suggest that it should be included in future studies.

The main limitation of this study is the risk of residual confounding from unmeasured confounders such as comorbidities and the current UTI symptom duration, which might affect antibiotic-prescribing decisions but were not recorded in the POETIC study.^15^ However, the POETIC study did not include patients with UTI symptoms lasting >2 weeks. Moreover, the authors recognise that the 2 days longer time to recovery among those who consumed no antibiotic might be so if longer follow-up was considered; however, the survival analysis median time to recovery as opposed to the mean would not be affected by extremely longer recovery durations. The POETIC trial included a follow-up at 3 months on UTI recurrence, but this was not considered for this study as the researchers were interested in the immediate recovery from uncomplicated UTI infection. Though antimuscarinic, antifungal, and analgesic use was adjusted for, binary indicators were used and did not consider the amount, duration, or time point these were consumed, as this information was not collected. Data from a randomised trial of a point-of-care test were used that could potentially affect antibiotic prescribing. However, the test results were not available to clinicians until approximately 24 hours after the initial consultation, the intervention had little overall effect on antibiotic use,^16^ and patients who were prescribed a new or different antibiotic after the initial consultation were excluded in the current analysis. Therefore, the authors think the trial intervention is unlikely to have biased the present findings. The exclusion of patients who changed antibiotics was also to clarify the definition of both exposure and recovery time, for example, antibiotics might be changed because of evidence of resistance, which could affect outcomes. A relatively small number of patients fell into this category, and the researchers feel that their exclusion is unlikely to have biased the findings. Finally, data on antibiotic consumption provided by patients were used, which may not be as accurate as medication adherence monitoring containers or measurement of antibiotic levels in blood or urine.^27^

### Comparison with existing literature

A recent Cochrane review on antibiotics efficacy for UTI commented on the lack of data to evaluate time to symptomatic recovery.^28^ In addition, the evidence from placebo-controlled trials is scarce. Only three such trials reported symptom duration in a review on the natural course of uncomplicated UTI in females up to November 2019.^29^ Their results showed that complete symptom recovery could occur in 18% (up to 3 days) to 54% (up to 6 weeks) of the placebo arm, with most improvement occurring in the first 9 days. This is consistent with the present finding, estimating that 50% of those who did not consume antibiotics would recover completely by day 9.

Moreover, antibiotics were found to be superior to no-antibiotic management strategies, such as painkillers, for uncomplicated UTI in females. While ∼40% of females recovered by day 4 with ibuprofen, ∼70% recovered with pivmecillinam.^30^ With a higher dose of ibuprofen ∼70% of females recovered by day 7 compared with 82% for fosfomycin.^12^ In addition, recovery time prolonged with diclofenac (4 days) compared with norfloxacin (2 days).^31^ No-antibiotic management may be suitable for females with mild to moderate symptoms, with caution about potential risk of pyelonephritis.^12^^,^^30^^,^^31^

### Implications for research and practice

The present study found an association between antibiotic consumption and shorter time to recovery in females with uncomplicated UTI in primary care, which is consistent with evidence from trials suggesting net overall benefit from antibiotics.^14^ However, many females with UTI symptoms make a good recovery without antibiotic treatment and only mild symptoms remain (Supplementary Figure S1). They may choose to delay antibiotic treatment and/or consider alternative treatments for their symptoms.^10^^,^^32^ The present results may help informing such a shared decision-making approach if patients were aware of the likelihood of recovering within 14 days without antibiotics. These strategies are particularly useful in younger patients and in those with lower risk for pyelonephritis^10^^,^^31^^,^^33^ and could lead to a reduction in antibiotic use and risk of resistance.^26^ Attention should focus on trying to identify these females, particularly as it is possible that some of them do not have bacterial infections (only 34% of the study group had culture-confirmed bacterial pathogen), or that their immunity are able to self-limit the infection.

There is also a need for further studies quantifying the risk and predictors of complications associated with UTI managed with and without antibiotics.

## Data

Data available on request from the author.
